# Population-Level Nutritional Well-Being: Nutrition Security and Equitability

**DOI:** 10.3390/healthcare11060817

**Published:** 2023-03-10

**Authors:** Kaydian S. Reid

**Affiliations:** Department of Nutrition and Public Health, University of Saint Joseph, West Hartford, CT 06117, USA; kreid@usj.edu

Nutrition is essential to sustaining the quality of life and a fundamental right of all people [1]. Yet, many nutrition practices, communications, research, and policies focus on the clinical approach with less emphasis on population-based health. For instance, the global obesity prevalence, a chronic disease pandemic, has challenged practitioners, food systems, and policymakers to shift their approach to include population-level initiatives. The shift is complex, interdependent, and constantly evolving, requiring a comprehensive approach by all stakeholders to ensure that the health and wellbeing of the public are maintained. Public health approaches to nutrition emphasize the importance of looking at the big picture when it comes to nutrition—not just focusing on individual dietary changes and nutrition education but also considering social and environmental factors that influence diet. Furthermore, public health and nutrition relationships involve exploring other areas such as health promotion, disease prevention, and population-level interventions to create healthier food environments for all community members. This relationship promotes and sustains the nutritional health of the population, which is the fundamental resource for local, national, and global communities’ social, cultural, environmental, and economic well-being [1,2,3].

The *Healthcare* Special Issue, “Nutrition and Public Health 2.0”, brings together research from scholars of different disciplines representing various regions of the world to understand achievements and current issues in nutrition and public health, including research, practice, policy, and communication across the lifespan. The articles selected are centered on two public health nutrition concepts: nutrition security and nutrition equitability.

## 1. Nutrition Security

Nutrition security ensures that individuals, families, and countries have physical, social, and economic access to sufficient, affordable, safe, and nutritious food that meets their dietary needs and preferences for an active and healthy life [4,5]. Nutrition security also includes the understanding, skillset, and available resources needed for individuals to make healthy dietary choices for themselves and their families. For example, Storz et al. [6] investigated the daily nutritional goals that were met and for which nutrients an insufficient or excessive intake occurred. The study population comprised adults from the United States, half of the sample consuming acidifying diets and the other half consuming alkaline-promoting diets. The results indicated that individuals that consumed alkaline-promoting diets met their nutritional goals compared to those who consumed acidifying diets. However, neither group met the standards for critical nutrients for bone health. These findings suggest that alkaline-promoting diets tend to meet the Dietary Guidelines for Americans Guidelines’ recommendations on some macronutrients. Another study by Albalawi et al. [7] examined the prevalence of micronutrients and arsenic levels among individuals in Saudi Arabia. The study reported that among the 14 micronutrients, adults in the sample were more deficient in vitamin D (64.3%). Additionally, one in five adults had an iron deficiency, and approximately 99% of the sample had normal arsenic levels. A cross-sectional stud by Al-Qahtani et al. [8] observed that children under two years of age and exclusively breastfed were at greater risk of vitamin D deficiency than those who were not exclusively breastfed. Vitamin D is vital for bone development, especially among young children. These articles suggest that population health should go beyond food security to nutrition security, where both nutritional values of food and systemic factors may determine intrapersonal nutritional status, while focusing on community-level access and quality. Systemic factors beyond the intrapersonal level of control are likely to influence dietary behaviors, nutrition, and health.

## 2. Nutrition Equitability

Nutrition equitability is an emergent concept that addresses the food system stakeholders and holds the stakeholders accountable to ensure accessibility and affordability of sufficient nutrition regardless of individuals’ social, environmental, and economic positions [9,10]. This concept is based on the idea that everyone should have fair access to the same types of quality nutrition, regardless of their circumstances. Al Banna and colleagues [11] investigated the association between nutrition literacy and healthy eating behaviors among Bangladeshi adults. The authors observed a positive relationship between healthy eating behavior and nutrition literacy. Health literacy intersects with health equity because those with lower health literacy are often at increased risk for adverse health outcomes due to limited understanding of health information, lack of access to health care, or poor patient–physician communication. In terms of public health, increasing nutrition equitability will help prevent malnutrition, deficiencies, and associated health problems, especially among children. It can also reduce the burden of many preventable diseases worldwide, from cardiovascular disease to metabolic disorders. Park et al. [12] conducted an explorative study at community childcare centers during the COVID-19 pandemic using an interactive cooking program with digital technology among children and staff. Their findings suggest that community childcare centers are ideal settings for healthy eating practices with the aid of digital technology to provide resources for marginalized populations. Equitability in interventions is essential for both nutrition and public health.

In summary, the 16 articles in the Public Health and Nutrition 2.0 Special Issue document that to achieve population-level nutritional well-being, nutrition equitability, and security [6,8] is essential. To do so, governments at all levels should strive to provide education and community-based interventions on healthy eating [13] and adequate resources for good nutrition [14,15,16], such as introducing subsidies for basic food items and ensuring fair distribution of food items to all areas of society regardless of their socioeconomic status. Each study presented in this Special Issue provides a greater understanding for public health and nutrition stakeholders to reconsider the level of intervention needed to address and reduce nutrition-related health outcomes effectively. We hope scholars—globally—will continue to expand our knowledge, understanding, practices, and policies to improve health outcomes across the lifespan regardless of socioeconomic economic position.
